# A benchmark of algorithms for the analysis of pooled CRISPR screens

**DOI:** 10.1186/s13059-020-01972-x

**Published:** 2020-03-09

**Authors:** Sunil Bodapati, Timothy P. Daley, Xueqiu Lin, James Zou, Lei S. Qi

**Affiliations:** 1 Department of Bioengineering, Stanford University, 450 Serra Mall, Stanford, 94305 USA; 2 Department of Statistics, Stanford University, 450 Serra Mall, Stanford, 94305 USA; 3 Department of Biomedical Data Science, Stanford University, 450 Serra Mall, Stanford, 94305 USA; 4 Department of Chemical and Systems Biology, Stanford University, 450 Serra Mall, Stanford, 94305 USA; 5 ChEM-H Institute, Stanford University, 450 Serra Mall, Stanford, 94305 USA; 6 Present Address: Affirm Inc., San Francisco, USA

**Keywords:** CRISPR screen, CRISPR knockoout, CRISPR interference, CRISPR activation, Benchmarking, Screen algorithms, Simulation

## Abstract

Genome-wide pooled CRISPR-Cas-mediated knockout, activation, and repression screens are powerful tools for functional genomic investigations. Despite their increasing importance, there is currently little guidance on how to design and analyze CRISPR-pooled screens. Here, we provide a review of the commonly used algorithms in the computational analysis of pooled CRISPR screens. We develop a comprehensive simulation framework to benchmark and compare the performance of these algorithms using both synthetic and real datasets. Our findings inform parameter choices of CRISPR screens and provide guidance to researchers on the design and analysis of pooled CRISPR screens.

## Introduction

Clustered regularly interspaced palindromic repeats (CRISPR) and the CRISPR-associated (Cas) proteins are a class of bacteria-encoded, RNA-guided, programmable DNA targeting and cleavage systems. Due to its programmable nature using customizable single-guide RNAs (sgRNAs), CRISPR-Cas has enabled powerful pooled screens to explore the function of genetic perturbations at a genome-wide scale. Taking the most commonly used CRISPR-Cas9 system as an example, the *Streptococcus pyogenes* Cas9 protein can complex with a 110-nucleotide (nt) sgRNA containing a 20-nt sequence that complementarily binds to the target DNA region and induces a double-stranded break (DSB). This cutting mechanism on the genomic DNA triggers host non-homologous end joining (NHEJ) or homology-directed repair (HDR) pathways, which typically leads to loss-of-gene function. In addition to editing, nuclease-deactivated Cas (dCas) molecules have been engineered by introducing silencing mutations to abrogate the nuclease activity. Fusing dCas molecules to transcriptional or epigenetic effector domains allows sequence-specific gene regulation for either gene activation (CRISPRa) or repression (CRISPRi).

The intriguing programmable RNA-guided DNA targeting feature of CRISPR-Cas allows it to be scaled up to target many genomic sites in parallel in one experiment. High-throughput DNA synthesis platforms can generate a library of oligos with various sequence features (hundred of thousands or even millions), with each oligo encoding a different sgRNA sequence, and thus a different DNA target. The oligo library can be cloned onto a lentiviral vector system to facilitate cellular delivery, integration, and expression. By transducing the sgRNA lentivirus pool into desired cell types (often at a multiplicity of infection much smaller than one to ensure less than one viral particle per cell), high-throughput, parallel loss-of-function genomic perturbations can be carried out to infer their functional relevance.

**Fig. 1:**
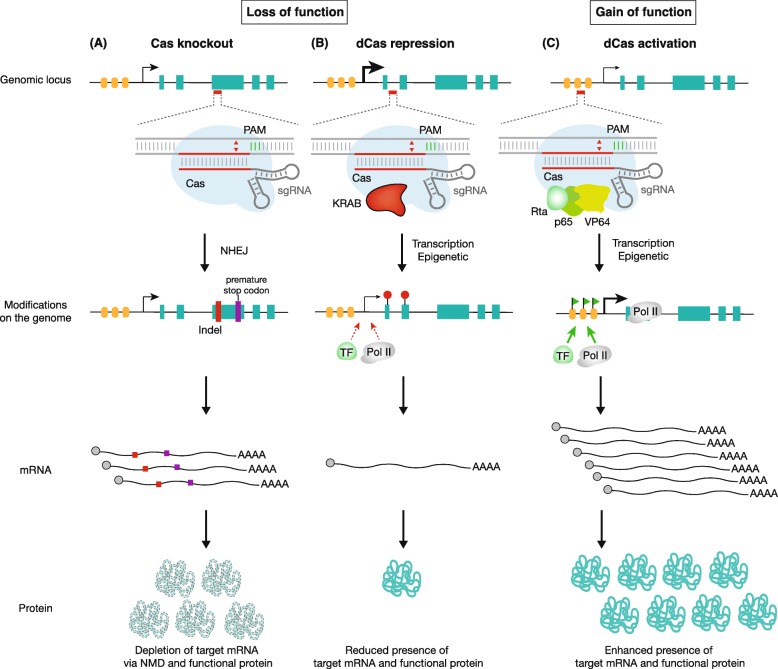
**a** Cas knockout is accomplished by targeted indel formation at a genomic site complementary to the sgRNA. An indel often results in a frameshift that causes premature stop codon formation that leads to non-sense-mediated decay (NMD) of target mRNAs or the generation of truncated non-functional proteins. **b** Programmable transcription repression can be achieved using dCas and repressor domains (e.g., KRAB) that are fused to the dCas. This complex either directly sterically hinders the recruitment of native transcription factors and RNA polymerase, or rewrites the nearby chromatin region to be more silencing to affect transcription status, leading to the reduced production of target mRNA and functional protein. **c** Programmable transcription activation can be achieved using dCas and activator domains (e.g., VP64, p65, and Rta) that are fused to the dCas. This complex recruits transcriptional machinery to the transcription start site of the desired gene, resulting in enhanced expression of the target mRNA and functional protein. NHEJ, non-homologous end joining; TF, transcription factor; Pol II, RNA polymerase II; PAM, protospacer adjacent motif

There are three main types of CRISPR-based genome perturbation technologies that researchers can use for pooled screens (Fig. 1). The CRISPR-Cas9 knockout (CRISPRko) method uses a catalytically active Cas9 to cut the target site close to the protospacer adjacent motif (PAM) within the sgRNA binding region, introducing a small indel (insertion/deletion) mutation (Fig. 1a) [1–3]. If the target site is in the exon or intron of the target gene, then this indel mutation could cause premature stop codon formation, which produces a non-sense transcript that will be degraded by non-sense-mediated decay. CRISPR inactivation (CRISPRi) uses a deactivated Cas9 (dCas9) to target the promoter or protein-coding region of the chosen gene and works in tandem with repressor domains (e.g., KRAB) to inhibit gene transcription [4, 5]. In CRISPR activation (CRISPRa), enhancer domains (e.g., VP64 or VPR) are attached to dCas9 and upregulates gene transcription when targeted to the gene promoter [5, 6]. CRISPRko and CRISPRi allow researchers to investigate loss of function when the gene is knocked out for complete loss of function or knocked down for partial loss of function, while CRISPRa allows researchers to investigate gain of function. The choice between CRISPRko and CRISPRi is up to the researcher. Typically, knockout is preferred because the signal is usually clearer. However, recent research has shown that gene function can be rescued after knockout via various mechanisms including truncated proteins, skipping of the edited exon, or compensation via gene paralogs [7, 8]. When choosing between knockout with CRISPRko and knockdown with CRISPRi, researchers should be aware of such issues.

There are many choices a researcher can make in the design of screens. First, the screen can either be arrayed or pooled. Arrayed designs can separate individual guides into different pools in the array. Thus, they obtain direct estimates of the genetic effects but are limited in their throughput (hundreds to a few thousands of genes, e.g., [9, 10]) and require high-throughput analysis (e.g., image-based) to measure the association with the desired phenotype [11]. This is an extremely difficult problem, as a multitude of batch effects and extrinsic noise can complicate the analysis (e.g., [12]). Pooled designs have a higher throughput, allowing for tens of thousands of genes and loci to be investigated, but because of the pooled design, all effects measured are relative to a baseline (usually estimated with negative control guides). Overwhelmingly, most applications use a pooled screen to identify possible hit genes for initial discovery and then use more direct experiments to measure the individual effects of each gene for validation. Therefore, we focus our attention on the design and analysis of pooled CRISPR screens.

In a typical screen, two populations are compared: the treated population, where the knockout, inhibition, or activation has taken effect and the population is selected for the phenotype; the control population, in which either no CRISPR-based effect has taken place or the effect is induced and the population is negatively selected for the phenotype. Proper choice of the control population is key to the success of the screen, yet which type of control population to choose depends on the type of screen. For example, in a screen for essential genes, those genes whose knockout or knockdown results in depletion of the corresponding guide RNAs, the treated population is taken to be the population several days after the inhibition or knockout occurs and the control population is often the initial (pre-knockout) population [13, 14]. On the other hand, in a screen for neurogenesis, we can take the treated population to be fluorescence-activated cell sorting (FACS)-sorted marker gene (e.g., beta-tubulin III)-positive cells and the control population to be marker gene-negative cells [15]. The normalized log fold change for each guide, which is the typical summary statistic of the screen, is computed by comparing the relative abundance in the treated population to the relative abundance in the control population, and normalizing such that the majority (mode) or average (mean) log fold change is equal to 0. This latter normalization assumes that the majority of genes have no effect and association with the phenotype. This assumption can be violated, for example, in [15], most genes had a negative effect on the phenotype. Such a situation needs to be checked prior to the analysis using the distribution of negative control guides (if included in the screen), and the analysis needs to be designed with such considerations in mind.

CRISPR technologies have been widely used to identify new druggable targets (e.g., for cancer) and to identify the genetic targets of existing drugs. Since the genes that are affected by drugs can go in both directions (resistance and susceptibility), both gain of function with CRISPRa and loss of function with CRISPRi and CRISPRko are used. To name a few examples, [16] used CRISPRko to identify genes whose loss is associated with resistance to vemurafenib, a therapeutic agent for melanoma; [17] used both CRISPRi and CRISPRa to identify microtubules as the target of rigosertib, a drug currently in stage 3 clinical trials [18, 19] used CRISPRa to identify genes and long non-coding RNAs (lncRNAs) that lead to resistance to the chemotherapeutic agent cytarabine; [20] used CRISPRko screen in human cancer cell lines to identify biomarkers for successful treatment; and [21] combined CRISPRko screens with RNA-seq data to associate gene fusion events with tumor susceptibility to anti-cancer drug treatment.

The majority of applications have targeted genes to identify the effect of their perturbation on a phenotype. However, CRISPR technologies can also be used to investigate the genetic function in finer detail. CRISPRko can be used to perturb protein coding sequences to identify the function of specific amino acids through dense tiling mutagenesis [22, 23]. Similarly, tiling screens using CRISPRi or CRISPRa can help to identify enhancer or functional non-coding domains [24–26]. The analysis of these screens require the researcher to take into account the gene or linear structure of the DNA [23, 27]. This is highly context specific, so we will not discuss the analysis of such screens.

Despite great utility, challenges still remain in CRISPR screens. Variability in guide RNA efficiency can complicate the analysis [28]. Varying gene effect sizes can result in a bias towards finding only genes with large effects [29]. Cell death from excessive cutting in high copy number regions can lead to false positives in CRISPRko screens [30–33]. These issues complicate the data analysis and often cause both false positives and false negatives. While many developed algorithms aim to address some of these issues, to a larger extent, it remains unknown how these algorithms impact the analysis and inference of biological conclusions and how these algorithms perform compared to each other.

In this paper, we aim to provide an overview and benchmark of the existing analysis algorithms for pooled CRISPR screens. In particular, we will systematically analyze how different algorithms impact analysis by benchmarking them in the face of some of the commonly faced issues listed above. Our goal is to provide a guide for researchers on experimental design and choices of algorithms and inform on what issues may arise and how these issues can be handled in the analysis. Since it is a fast-moving field, we may not cover all possible issues and algorithms. We benchmark algorithms on both simulated data, where the ground truth is known, and real data, where the ground truth is not entirely known but must be assumed.

## Algorithms summary

Below are the brief overviews of the existing algorithms commonly applied in the analysis of CRISPR knockout, activation, and inactivation screens. Each algorithm section includes the specific purpose the algorithm was designed for and a high-level overview of the math used to perform the analysis. A summary of the algorithms is given in Table 1. Some algorithms work directly on the count data, while others rely upon the existing tools for count analysis to obtain log fold changes for each guide, and then combine information across guides to perform analysis at the gene level.

**Table 1:** Algorithms for analyzing CRISPR-pooled screen data

Algorithm	Original purpose	FDRs?	Guide inefficiencies?	Negative controls?	Single or multiple screens?
RSA	RNAi	No	No	No	Single
MAGeCK RRA	CRISPRko	Yes	No	Yes	Single
HiTSelect	CRISPRko & RNAi	Yes	Yes	Yes	Single
MAGeCK MLE	CRISPRko	Yes	Yes	Yes	Both
BAGEL	CRISPRko essentiality	Yes	No	Yes	Single
CERES	CRISPRko in cancer	Yes	Yes	No	Multiple
CRISPhieRmix	CRISPRi/a	Yes	Yes	Yes	Single

### Redundant siRNA activity

Redundant siRNA activity (RSA) [34] is designed to identify important genes in RNA interference (RNAi) loss-of-function screens. RSA works by initially ranking all targeting guides by decreasing log fold change between the initial condition and final condition. The algorithm then assigns a *p* value to each gene using an iterative hypergeometric distribution formula that measures the statistical significance of a gene having highly ranked guides, assuming that under the null distribution, the ranks are uniformly distributed. Only the rankings of the guides, not the magnitude of the log fold change, are used in computing the *p* value. This approach allows for rare off-target guides with high effect sizes to be deprioritized compared to guides that all perform around the same. As output, RSA returns an ordering of genes ranked by essentiality but not their associated *p* values.

### MAGeCK robust ranking algorithm

Li et al. proposed robust ranking algorithm (RRA) [35] as one of the first algorithms specifically designed for CRISPR knockout screens. RRA, unlike RSA, takes raw sgRNA read counts to perform its analysis. With the counts, RRA fits a negative binomial model to test whether the initial sgRNA counts vary significantly from the final condition’s counts, in a similar manner as RNA-seq differential expression algorithms such as DESeq2 [36]. The resulting guide-level *p* values are combined at the gene level using a modified robust ranking algorithm. MAGeCK RRA returns a list of genes with corresponding estimated false discovery rates (FDRs) in the case of either a loss-of-function or gain-of-function screen.

### HiTSelect

Diaz et al. released HiTSelect in 2014 [37] to handle several issues in modern analysis of RNAi and small hairpin RNA (shRNA) screens. In particular, they use a random effects model to account for variance in sequencing depth and a multi-objective optimization to identify genes with multiple active sgRNAs (or shRNAs). First, a measurement of the guide activity is computed by calculating the log fold change for each guide by the ratio of the relative abundance of the guide in the treated population and the relative abundance of the guides in the control population. The algorithm then attempts to use a dual objective function to rank genes based on maximizing genes that have a large effect on the phenotype and genes having a large fraction of the guides being labeled as “active” versus not active. By maximizing these two quantities, the algorithm controls for variation of sequencing depth as well as downgrades guides that may have random off-target effects. HiTSelect returns a ranked list of genes and estimated FDR. HiTSelect is available only through a GUI interface, and therefore, we were unable to perform large number of simulations with the software. We are unfortunately unable to comment on the performance of HiTSelect.

### MAGeCK maximum likelihood estimation

In 2015, Li et al. [29] published the maximum likelihood estimation (MLE) module for MAGeCK to address the challenge of estimating gene effects across CRISPRko screens that spanned multiple conditions (i.e., different cell lines or drug treatments), as well as to explicitly incorporate variable sgRNA knockout efficiencies. Similar to RRA, MLE takes the raw counts of sgRNA in the initial condition and final condition, but it also requires a design matrix to specify which counts come from specific condition. MLE extends the work of RRA by fitting a negative binomial generalized linear model with log-link to guide level counts. It then fits a coefficient at the gene level to compute the gene effect sizes and *p* values. This allows information to be pooled across many screens, while returning gene effect sizes on a per-screen basis. Like MAGeCK RRA, MAGeCK MLE returns a list of genes with FDR for both the loss-of-function and gain-of-function case. In addition, it returns estimates of gene effect size, something which most algorithms avoid.

### BAGEL

In many cases, CRISPR screens are known to produce specific effects. This prior information can be used to inform the analysis. For example, in the case of gene essentiality screens, a well-studied phenotype, prior information can be obtained from previous screens for gene essentiality. Hart and Moffat [38] designed the BAGEL algorithm to take advantage of this prior information. BAGEL uses prior distributions of the null and positive effects to calculate Bayes factors for each gene, which can then be used to rank genes. However, this only works if researchers have access to good prior information on the phenotype, which limits the broad applicability of this method.

### CRISPhieRmix

Daley et al. developed CRISPhieRmix [28] to address issues in the analysis of CRISPR activation and inactivation screens, specifically the issue of variable guide efficiency that are ubiquitous in these screens. CRISPhieRmix takes as input the log fold changes of the sgRNA from the initial condition to the final condition, typically estimated by standard count software such as DESeq2 [36] or edgeR [39], and then fits a hierarchical mixture distribution, assuming that the guides for hit genes can follow a mixture distribution. The estimated FDRs are calculated by first calculating the posterior probability each gene is null, then marginalizing over all possible mixtures, and finally obtaining FDR through an empirical estimator of the marginal false discovery rate. CRISPhieRmix returns a ranked list of genes and estimated FDR, but not gene effect sizes as they are non-identifiable in the hierarchical mixture model used by CRISPhieRmix.

### CERES

Cancer cells typically exhibit large copy number variations (CNVs). Using Cas proteins to cut sites with high copy number can introduce gene-independent effects due to cell death from a cell response to DNA damage. Meyers et al. proposed CERES [31] to computationally correct for this effect. To remove this bias, CERES compares each gene’s guides across all cancer cell lines tested to normalize for cell line-specific changes. CERES then fits a model to the experimental data by fitting an alternating least squares regression for each gene across all cell lines. However, CERES is only applicable to experiments involving multiple screens across several cell types with known copy number profiles; otherwise, the copy number effects are non-identifiable. Because of this, we did not apply CERES in our benchmarking study.

### JACKS

JACKS [40] is a Bayesian-based method primarily designed to pool information from multiple screens to improve inference across all screens. JACKS decomposes the guide-level log fold changes into a product of treatment-specific effects and guide-specific effects. Multiple screens from the same library allow JACKS to better model the guide-specific effects and improve inference on the treatment-specific effects. Though JACKS is applicable to single-screen experiments, it is primarily designed for multiple experiments where statistical pooling can help inference across all screens and therefore was not included in our single-screen benchmarking.

### *t* test

As a simple baseline, we also apply a standard *t* test. Specifically, we compute moderated log fold changes for all guides together using DESeq2 [36], then we apply a pooled two-sided *t* test for each gene by comparing the gene-targeting guides to the negative control guides. To ensure the applicability of the *t* test even when there is one guide per gene, we use the pooled variance computation, and this should be conservative because the pooled variance will be larger than if the variance were computed separately. We then apply Benjamini-Hochberg [41] to compute FDRs for all genes.

### Simulation framework

When comparing algorithm performance, it is important to understand the ground truth of the system that is being analyzed. Unfortunately, in many biological experiments, the ground truth is not known. In contrast, simulations have a well-defined ground truth, making them a useful tool for algorithm comparison. We developed a simulation framework to model the read counts of a loss-of-function CRISPR essentiality screen. Parameters that we investigated in our simulation include gene effect sizes, the number of guides per gene, guide efficiency, sequencing depth, and the number of control guides. To accurately compare these CRISPR algorithms to each other, we simulated different CRISPR screens by varying the parameters and compared which algorithms performed better under which conditions.

Our simulations assume that the effect of the phenotype is negative, as it is for loss-of-function or essentiality screens. Screens exploring gain of function, e.g., [15], will have effects in the positive direction. Our previous experience with such screens leads us to believe that there are common issues in the analysis of both types of screens. However, screens that investigate the effects in both positive and negative directions, such as in drug susceptibility/resistance screens, may involve more complex issues. We believe that our simulation can still provide some insight into the design of such screens.

## Simulation parameters

### Gene effect size

We assume that non-essential genes have an average effect of 0. The baseline essential gene effects are simulated from a truncated normal distribution of mean of − 1.2 log fold change (towards the negative end), variance of 5, and upper bound of − 0.7. The upper bound limits our analysis to only genes with a sufficiently high effect size, and the truncation allows for a long tail of effect sizes that we commonly observe in CRISPR screens. Individual guide effects are drawn from a normal distribution with a mean equal to the corresponding gene effect size and variance of 0.1. To simulate the varying gene effect sizes, we multiplied the baseline gene effect sizes by constants varying from 0.2 to 1. This will lower the average gene effect size as well as the upper bound of the effect size and allow us to investigate the cases where the signal is low compared to the inherent noise of the screen.

### Guide binding efficiency

Recent studies have shown that not only do sgRNAs have variable effects, but these guides may also have individual probabilities of either binding and having an effect or not binding and having zero effect. This may be due to local sequence effects or epigenetic effects [42]. We included a binding efficiency parameter to model the probability that a given guide will bind to the target site and cause a gene effect and varied this parameter from 1 (all guides have an effect) to 0.2 (few guides have an effect). This range covers previously estimated binding efficiency for CRISPRko (> 0.9, [43]), CRISPRi (0.7 to 0.4, [44]), and CRISPRa (0.7 to 0.2, [15] and unpublished data). We then sampled the binding effects from a Bernoulli distribution with probability of success equal to the binding efficiency. A 0 will mean we sample the guide effect from the null distribution, and a 1 will mean we sample the guide effect from the gene effect distribution (as detailed in the previous section).

### Sequencing depth

Low sequencing depth often leads to a low signal to noise ratio. To identify how robust algorithms are to various sequencing depths, we varied the average sequencing depths from an average of 1, 5, 10, 25, 50, 100, and 200 reads per guide.

### Number of guides per gene

An individual guide may have various biological reasons for not binding to a region of the DNA. To overcome these effects, increasing the number of guides per genes will allow for a high probability of identifying true signal in the resulting counts. We performed simulations with 1, 2, 4, 5, 10, and 20 guides per gene.

**Fig. 2:**
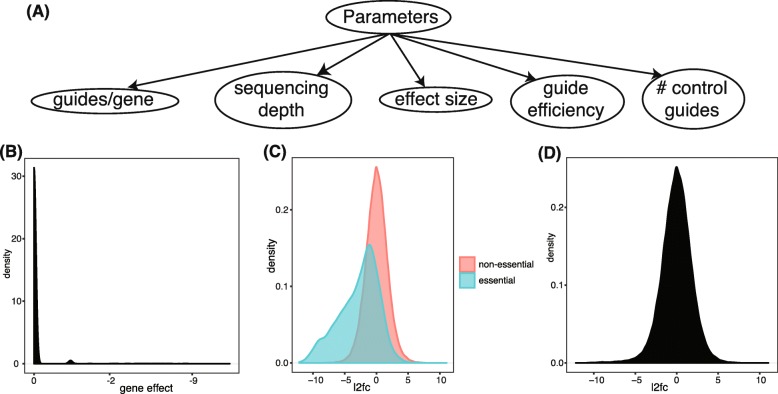
**a** An overview of the underlying screen parameters that we investigate. **b** Smoothed density plot of the baseline gene effect size. The vast majority of genes have no effect, while the essential genes with some effect have a long tail. **c** Example of a smoothed density plot of guide-level log2 fold changes, separated by unknown label. **d** Smoothed density plot of all guides with the labels hidden, as this is the distribution that must be deconvolved the analyst

To ensure our simulations reflected the results of a real screen, we designed it using a set of existing CRISPRko screens as a starting baseline [45]. For each simulation replicate, the initial guide abundance is sampled from a gamma distribution with shape and scale both equal to 1 (ensuring an average abundance of 1). This is a guide-specific variability that is constant across both the initial and the treated population and across replicates. This therefore represents technical effects. We then sample the initial population counts from independent Poissons with mean equal to the initial abundance times the sequencing depth times independent gamma(1,1) noise. To calculate the treated population abundance, we multiplied the initial guide abundances and the gene-level log fold change. We then normalized the treated population abundances to have a mean equal to 1 and multiplied by the sequencing depth and independent gamma(1,1) noise and sampled the treated counts from independent Poissons with means equal to the calculated guide abundances. We set the number of essential genes equal to 600 and the number of non-essential genes equal to 18,000, so that approximately 3*%* of genes are true positives. For baseline parameters, we set the number of guides per gene equal to 5, the gene effect size equal to 0.8 × 1.2, guide efficiency equal to 1, 500 control guides, and average sequencing depth equal to 100. To verify this simulation, we ensured that the outcome distribution was similar to the outcomes of real gene essentiality screens (Fig. 2, Additional file 1: Figure S1-6).

For each simulation, we applied RSA, MAGeCK RRA (RRA), MAGeCK MLE (MLE), and CRISPhieRmix on the simulated counts. As a baseline, we also applied DESeq2 to the guide-level counts and for each gene applied a *t* test against the log fold changes of the control guides. To ensure applicability to all cases, we used a pooled *t* test. This should be conservative as the variance should be at least as small as that of the combined population. FDRs were then computed by using the Benjamani-Hochberg procedure. HiTSelect was excluded from this analysis due to the fact that it could only be operated by a graphical user interface, making the programmatic testing under hundreds of parameters unfeasible. CERES was not an appropriate algorithm to use, given that it is primarily used for CNV effect control, which was not modeled in the simulation. We also excluded BAGEL, given that it requires prior knowledge of what different distributions should look like before analysis. We assumed that most CRISPR experimentalists are using algorithms to investigate genes with unknown phenotypes, which would complicate the application of BAGEL.

The full code for the benchmarking simulations is publicly available at [46].

## Results

For the benchmarking analysis, RSA,RRA, MLE, CRISPhieRmix, and the *t* test were compared. For each simulated or real dataset, the corresponding gene ranking output for each algorithm was then compared with the known essential genes. Area under the curve (AUC) for precision-recall (PR) curves were calculated and reported for each algorithm. RRA, MLE, CRISPhieRmix, and the *t* test are algorithms that also report a false discovery rate (FDR). We used a cutoff of 0.1 and computed the empirical FDR for genes below the cutoff. For algorithms that did not return any genes with an estimated FDR of 0.1 or below in any of the 3 replicates we used, we removed the data point from the graph.

### Number of guides per gene

**Fig. 3:**
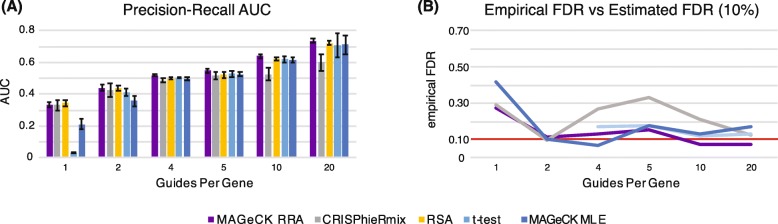
**a** Area under the precision-recall curve (PR-AUC) for each algorithm with increasing guides per gene. The bar heights and error bars are calculated respectively as the mean and standard deviation from three replicate simulations. **b** Empirical false discovery rate at an estimated false discovery rate of 0.1. Plotted values are the average over three simulations. Missing values mean that the algorithm did not identify any significant genes at an FDR of 0.1

We first investigated the effect of the number of guides per gene on the analysis. We performed three replicates of 1, 2, 4, 5, 10, and 20 guides per gene. We found that the performance of all algorithms is severely deteriorated when one or two guides per gene is used, particularly the *t* test and MAGeCK MLE (Fig. 3). At one guide per gene, the empirical false discovery rate was exceedingly high for all algorithms, while at two guides, it becomes acceptable. This indicates that multiple guides per gene are absolutely necessary for proper control of the FDR. At 20 guides per gene, all algorithms are better able to distinguish between the null and positive genes, though the cost of 20 guides can be high for the improved performance. Our simulations indicate that four guides per gene is likely the minimum number of guides per gene needed. If the genes or the phenotype under investigation has low signal, then more guides will be necessary.

### Number of control guides

We next investigated the effect of the number of control guides. We found that increasing the number of control guides does not affect the precision-recall AUC for all algorithms but CRISPhieRmix (Additional file 1: Figure S7). This makes sense, since only CRISPhieRmix uses the control guides in the ranking step. However, at least 300 control guides are needed to effectively control the empirical FDR. Otherwise, too many false discoveries will overpower the true discoveries, which will then complicate the downstream analysis. With no control guides, we found that MLE, RRA, and especially CRISPhieRmix tend to have high empirical FDRs, while the *t* test is no longer applicable. We suggest the inclusion of control guides is necessary, and more are needed for more complicated investigations and experiments. High signal to noise experiments will require fewer control guides, perhaps only a few hundred, while low signal to noise experiments will require more. Since our simulations would be considered the former case, at least 300 are needed.

### Sequencing depth

**Fig. 4:**
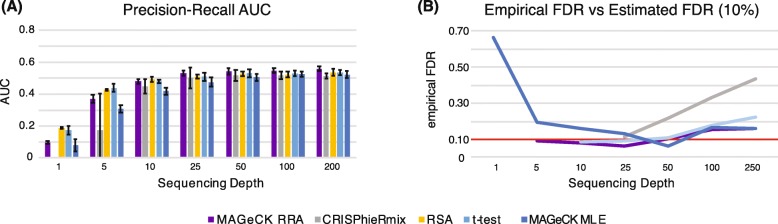
**a** Area under the precision-recall curve (PR-AUC) for each algorithm as a function of average sequencing depth. The bar heights and error bars are calculated respectively as the mean and standard error from three replicate simulations. **b** Empirical false discovery rate at an estimated false discovery rate of 0.1. Plotted values are the average over three simulations. Missing values mean that the algorithm did not identify any significant genes at an FDR of 0.1

The suggested sequencing depth for CRISPR sequencing experiments is one to two hundred reads per sgRNA [47]. We tested this claim by varying the sequencing depth from an average of one guide per gene per condition and replicate to two hundred guides per gene. We found that the performance of most algorithms plateaued at 25 reads per gene, both in terms of precision-recall and empirical FDR (Fig. 4). Our simulations indicate that at a constant sequencing depth, higher performance will usually be obtained with 20 guides per gene at a depth of 25 reads per guide than at 5 guides per gene at 100 reads per guide. More guides per gene gives the algorithms a better chance to model and account for both biological and technical variability. Higher sequencing depth, on the other hand, only accounts for sampling variability. The former two sources of variation tend to be larger than the latter.

### Gene effect size

To understand the effect of lower signal, we repeated the simulations with gene effect sizes multiplied by 0.2, 0.4, 0.6, 0.8, and 1 to simulate the performance under lower signals. We found several notable observations (Additional file 1: Figure S8). First, the MAGeCK RRA seemed to be the most robust with respect to smaller effect sizes, although all algorithms exhibit degraded performance with smaller effect sizes. At the smallest effect size, all algorithms have extreme difficulty in distinguishing the null from the non-null genes. Finally, with smaller effect sizes, it is more difficult to control the false discovery rate. Thus, in complex investigations, it may be beneficial to use more control guides in the experiment.

### Guide binding efficiency

**Fig. 5:**
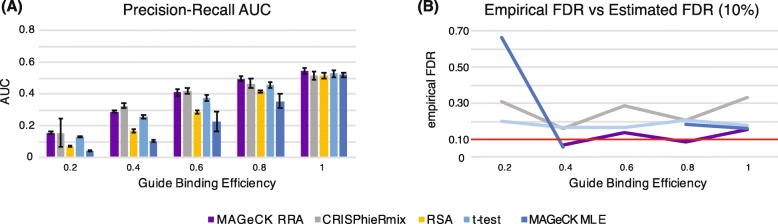
**a** Area under the precision-recall curve (PR-AUC) for each algorithm as a function of increasing guide binding efficiency. Lower guide binding efficiency implies that more guides from essential genes look like the null distribution. The bar heights and error bars are calculated respectively as the mean and standard error from three replicate simulations. **b** Empirical false discovery rate at an estimated false discovery rate of 0.1. Plotted values are the average over three simulations. Missing values mean that the algorithm did not identify any significant genes at an FDR of 0.1

We next investigated the effect of variable guide efficiency on the algorithms. In CRISPRi and CRISPRa screens, more so than in CRISPRko screens, variable guide efficiency is a major issue. To simulate this, we applied a random Boolean mask to the guides. If the Boolean mask is 1, then the guide effect is drawn from the appropriate effect distribution, and if the Boolean mask is 0, then the guide effect is drawn from the null distribution. We simulated the probability of 1 in the mask as 0.2, 0.4, 0.6, 0.8, and 1. As expected, all algorithms had worse performance with decreased guide efficiency (Fig. 5). Indeed, when only one fifth of the guides work, then the probability that a gene has no working guides is 33%, and no algorithm can identify such genes when this occurs. The algorithms that performed the best in the presence of guide variability were CRISPhieRmix and MAGeCK RRA. The former is not unexpected, since it is the only algorithm that is specifically designed for this situation. However, the latter is surprising because MAGeCK MLE shows highly degraded performance with decreased guide efficiency. It may be because the robust rank aggregation that MAGeCK RRA uses is more robust to variable guide efficiency than the GLM that MAGeCK MLE uses.

## TKO results

We used the well characterized Toronto KnockOut (TKO) library [45] to compare the different algorithms against each other. The TKO CRISPR knockout screen targeted 17,230 genes in 6 different cancer cell lines, with an average of 5 guides per gene, but with the vast majority of genes having 6 guides. The library contains 3 types of control guides, non-human gene-targeting guides (EGFP, LacZ, Luciferase), guides targeting random regions of Chr10, and guides targeting high repeat (greater than 20 per guide) regions of Chr10. We used the 684 genes identified by Hart et al. [48] as commonly essential in all cell lines as known true-positive genes. One note to consider is that in this study, Hart et al. found 1580 “core” essential genes, but only 684 genes were observed as significantly depleted in every cell line. Their analysis benefited from their prior information obtained from RNAi screens [49]. We assume that the algorithms that we are testing do not have such prior information and should show worse performance than their algorithm, BAGEL [38]. Therefore, it is reasonable to assume that all commonly essential genes are true positives. However, there may be more true positives in each screen. Therefore, we evaluated the performance assuming that the commonly essential genes are the only true positives with all other genes null. This should overestimate the FDR and underestimate the PR-AUC. We also evaluated the performance assuming that the commonly essential genes are true positives only the genes identified as commonly non-essential are the only true nulls (essentially ignoring all other genes), and this will underestimate the FDR and overestimate the PR-AUC. We tested the performance when the non-human guides were used as controls and when the randomly targeting guides were used as control. The algorithms that do not use the control guides to rank genes will be more robust to misspecification or mischaracterization for the control guides. For the ones that do, this will test their robustness.

We found that MAGeCK RRA had fairly consistent high performance. Sometimes, CRISPhieRmix and the *t* test performed better, but that only occurred when chr10Rand was used for the control guides. Because there are many more chr10Rand controls than non-human, the distribution of the combined set is primarily determined by the distribution of the chr10Rand. The *t* test and CRISPhieRmix performed poorly when the non-human guides were used as controls, particularly CRISPhieRmix. This indicates care must be taken when applying these algorithms, and the user must check that the central peak of the control guide log fold change distribution has a similar shape and the same mode as the distribution of the gene-targeting guide log fold changes. This checks that the distribution of the control guides reflects the distribution of the null genes, under the assumptions that most genes are null. We found that RSA tended to perform poorly on real CRISPR screening data, which is not surprising since it is designed for a different technology with different patterns and biases.

We see relative concordance of the performance of algorithms on the TKO dataset as compared to the performance on our simulations (Table 2). RRA performs the most consistently, with CRISPhieRmix sometimes outperforming based on the controls used. Surprisingly, the *t* test performs well in most cases. Although, like CRISPhieRmix, it is sensitive to controls and can perform extremely poor in some cases.

**Table 2:** Area under the precision-recall curve (PR-AUC) for all algorithms applied to TKO datasets, using the combined non-human- and Chr10Rand-negative control guide RNAs

	HCT116_1	HCT116_2	DLD1	HeLa	RPE1	GBM	Average rank
MAGeCK RRA	**0.449**	0.505	0.508	**0.424**	0.450	0.461	**2**
MAGeCK MLE	0.404	0.476	0.372	0.395	0.402	0.346	3.7
CRISPhieRmix	0.459	0.435	0.032	0.391	**0.502**	**0.535**	2.7
RSA	0.346	0.415	0.439	0.340	0.389	0.368	4.5
*t* test	0.431	**0.535**	**0.57**	0.383	0.474	0.528	2.2

Each column corresponds to a specific cancer type. The average rank is calculated by taking the rank (1 to 5) for each screen and taking the arithmetic mean. The highest PR-AUC for each dataset is highlighted in bold

We next looked at the trade-off of sequencing depth versus the number of guides. First, we took the genes that had 6 guides, then we subsampled both the number of guides and the sequencing depth to keep the total sequencing depth for 3, 4, 5, and 6 guides. For example, at 3 guides per gene, we left the counts unchanged, while for 6 guides, we sampled the count of each guide as a binomial random variable with *n* equal to the original count and *p*=0.5. We did this to all libraries, applied MAGeCK RRA to all count files, and measured the performance in terms of both PR-AUC and ROC-AUC (Additional file 1: Figure S9). In all libraries, the ROC-AUC strictly increased as the number of guides per gene increased. This indicates that increasing the number of guides per gene (at the same sequencing depth) helps to better rank the true-positive genes higher. However, for the PR-AUC, the performance is strictly increasing for only one library. For three of the libraries, the performance peaks at five guides per gene, and for two, it decreases as the number of guides increases. This indicates that at a lower sequencing depth, it is harder to choose the correct cutoffs to maximize discovery, despite the fact that the positive genes are generally ranked higher.

## Discussion and perspective

### Recommendations for experimental design

Our simulations indicate that sequencing depth is a smaller factor than previously suggested [47]. We believe, based on our simulations and experience, that it would be better to use more guides at lower sequencing depth than to sequence few guides at a higher sequencing depth. Similar to suggestions in RNA-seq studies that suggest more replicates at higher coverage [50], more guides allow for powerful statistical methods that can incorporate technical and biological variability into their estimates, which in turn improves power. In contrast, increased sequencing depth only accounts for sampling variability, which we found to be marginal at 25 or more reads per guide. The number of guides needed depends on the phenotype under investigation and how strong the researchers expect the effect size will be. Smaller effect size means more guides and higher sequencing depth will likely be necessary. Additionally, we expect that as guide design improves to increase the on-target effects and decrease off-target effects (e.g., [51], [52], or [53]), the need for more guides per gene can be mitigated. We have made our simulation framework available to researchers so that they can perform simulations themselves and help to determine what is the optimal design for their experiment.

A surprising result that we found was the reasonable effectiveness of a simple *t* test. However, we used the log fold changes computed by the count analysis software DESeq2, which normalizes the log fold changes. This will handle most of the bias and variance introduced by the sampling nature of the count data. Unfortunately, the *t* test does not work without suitable control guides. We have previously found reasonable performance by combining the DESeq2 or edgeR guide-level *p* values at the gene level using Stouffer’s method [54] (unpublished data). Stouffer’s method rewards consistent signal across a majority of guides, in contrast to Fisher’s method which puts more reward on a single strong signal and can overestimate *p* values in the presence of outliers [55]. Indeed, we believe that any reasonable method will be able to identify the top genes, sometimes called the Pareto principle or 80:20 rule [56]. It is the genes with small effect sizes that are difficult to identify, which can be critical for researchers because of possible synergistic effects with some of the top genes. For example, in our previous research [15], we found several combinatorial interactions of high-effect size hit genes with low-effect size hit genes for efficient neuronal trans-differentiation using paired CRISPRa screening following a pooled direct CRISPRa screen. Improvement in the identification of these genes is needed, as these are the most difficult to find.

For downstream analysis, we suggest that in most cases, researchers should default to using MAGeCK RRA for the analysis. Our simulations suggest that it is robust and performs well in all cases. It is also well maintained, with active improvements, a thorough manual, and tutorial videos so that even users without extensive computational experience can use it. We expect that it will run reasonably well in most cases. When the screen is expected to have high variable guide efficiency, such as in CRISPRi or CRISPRa screens that investigate complex phenotypes, RRA has difficulty in finding hit genes, sometimes returning no hit genes in practice. In this case, we suggest CRISPhieRmix, as it is the only method that takes this issue into account. Although, its dependence on good controls is limiting and should be checked before use. When multiple screens are available on multiple cell types or cell lines and researchers want to identify both common hit genes and cell type-specific hit genes, then MAGeCK MLE, JACKS, and CERES are good options.

As we have shown, even simple methods can perform reasonably well in analyzing CRISPR screens. Because of this, we believe that the most promising area for future research is in identifying issues specific to the experimental design of CRISPR-pooled screens. The recent work in identifying biases such as copy number-associated effects [30–32], structural rearrangement effects [33], and bottleneck effects [57] is exemplary of promising directions. We believe that there are further questions to be answered: for example, Are such biases generalizable to CRISPR interference and CRISPR activation screens? Are there possible improvements to the experimental design that researchers could make to minimize bias and maximize signal to noise? How general are these biases to CRISPR systems not based on the Cas9, or even more specifically the *Streptococcus pyogenes* Cas9, protein?

## Supplementary information

**Additional file 1** Supplementary figures.

**Additional file 2** Review history.
