# Supplementary material for: A benchmark of algorithms for the analysis of pooled CRISPR screens
Source: Genome Biol. 2020 Mar 9;21:62. doi: 10.1186/s13059-020-01972-x (PMC7063732; doi:10.1186/s13059-020-01972-x)
Supplement: Supplementary file 1 — Additional file 1 Supplementary figures. [file 13059_2020_1972_MOESM1_ESM.pdf]

A benchmark of algorithms for the analysis of pooled CRISPR screens

Sunil Bodapati[1,*], Timothy Daley[1,2,*], Xueqiu Lin[1], James Zou[3,**], and Lei S. Qi[1,4,5,**]

[1]Department of Bioengineering, Stanford University

[2]Department of Statistics, Stanford University

[3]Department of Biomedical Data Science, Stanford University

[4]Chemical and Systems Biology, Stanford University

[5]ChEM-H Institute, Stanford University

[6]To whom correspondence should be addressed to: stanley.qi@stanford.edu

[*]These authors contributed equally

[**]These authors jointly supervised this work

Supplementary Figures

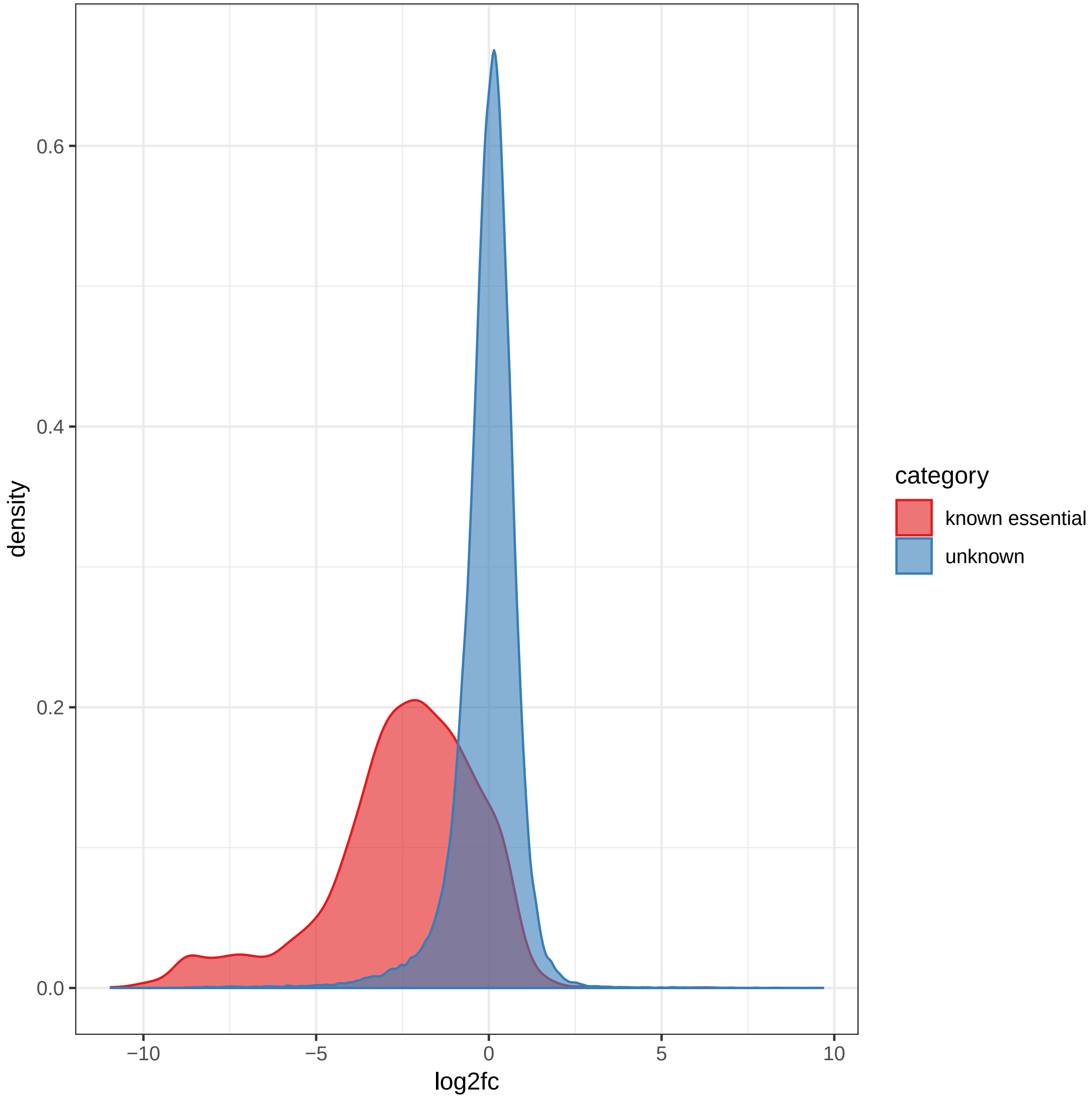

Fig S1: Distribution plots of guide-level $\log 2$ fold change for guides targeting known essential genes (red) and all other guides in the DLD1 cell line from Hart et al (2015).

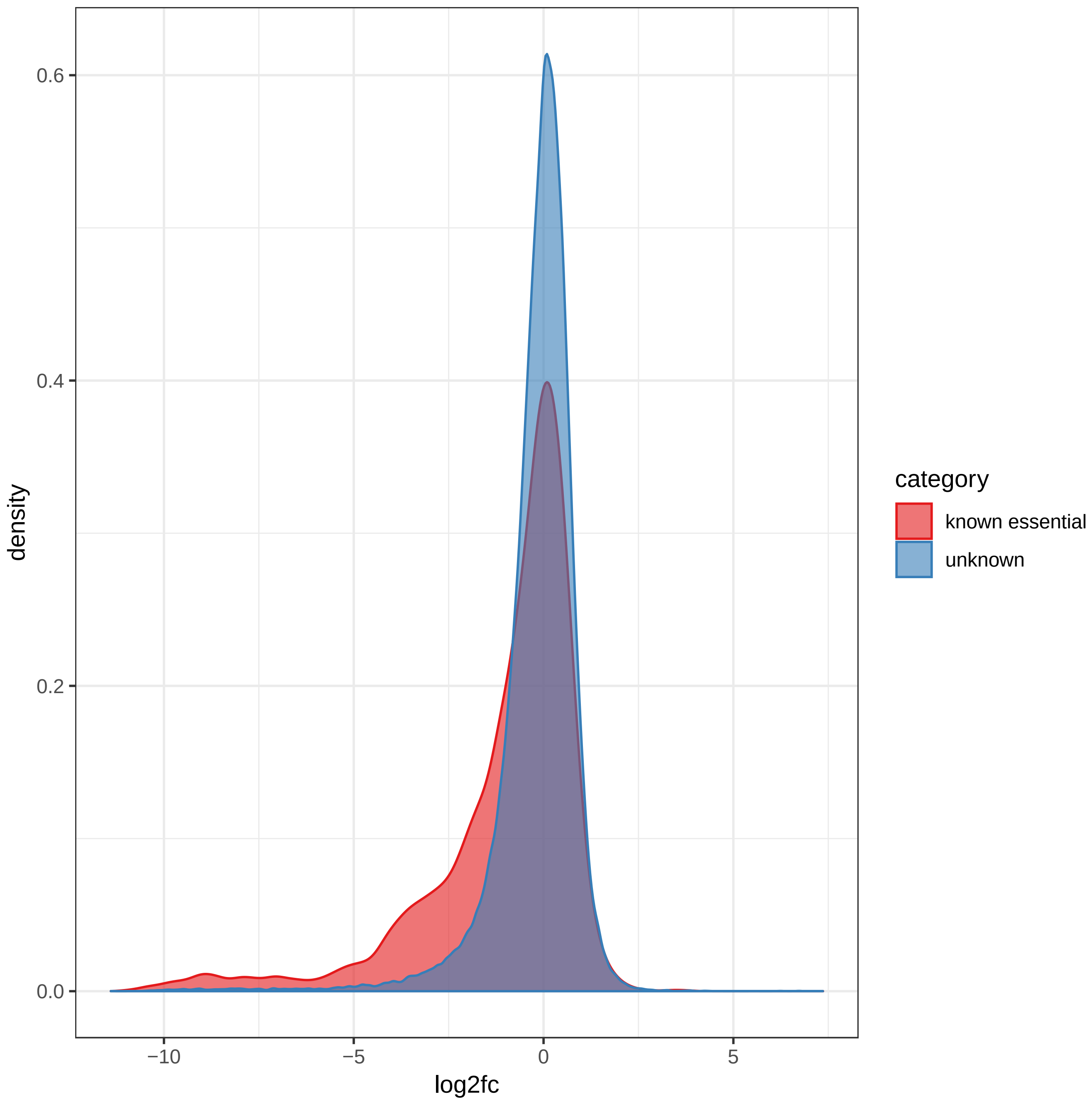

Fig S2: Distribution plots of guide-level $\log 2$ fold change for guides targeting known essential genes (red) and all other guides in the GBM cell line from Hart et al (2015).

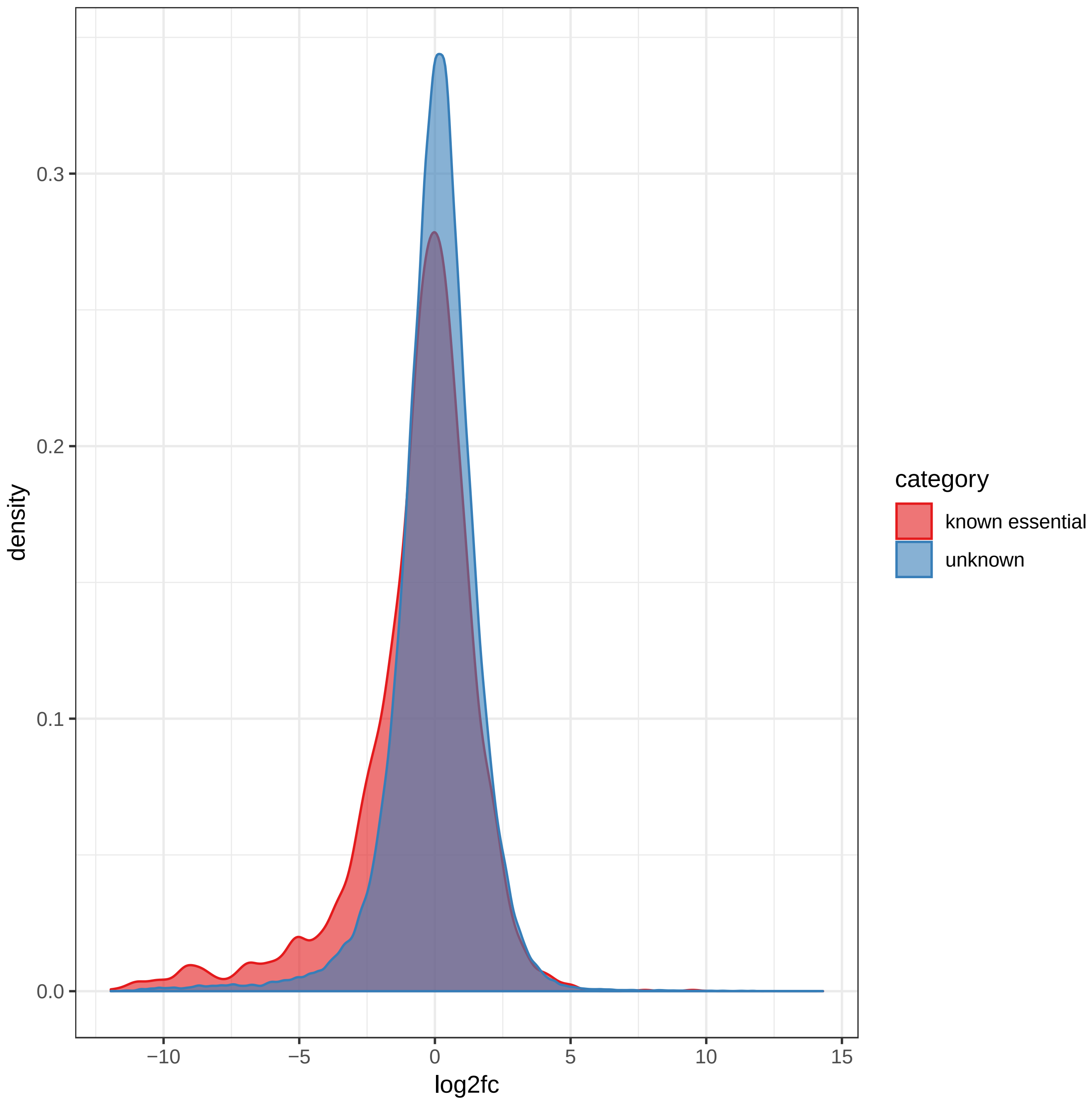

Fig S3: Distribution plots of guide-level $\log 2$ fold change for guides targeting known essential genes (red) and all other guides in the HCR116 cell line (replicate 1) from Hart et al (2015).

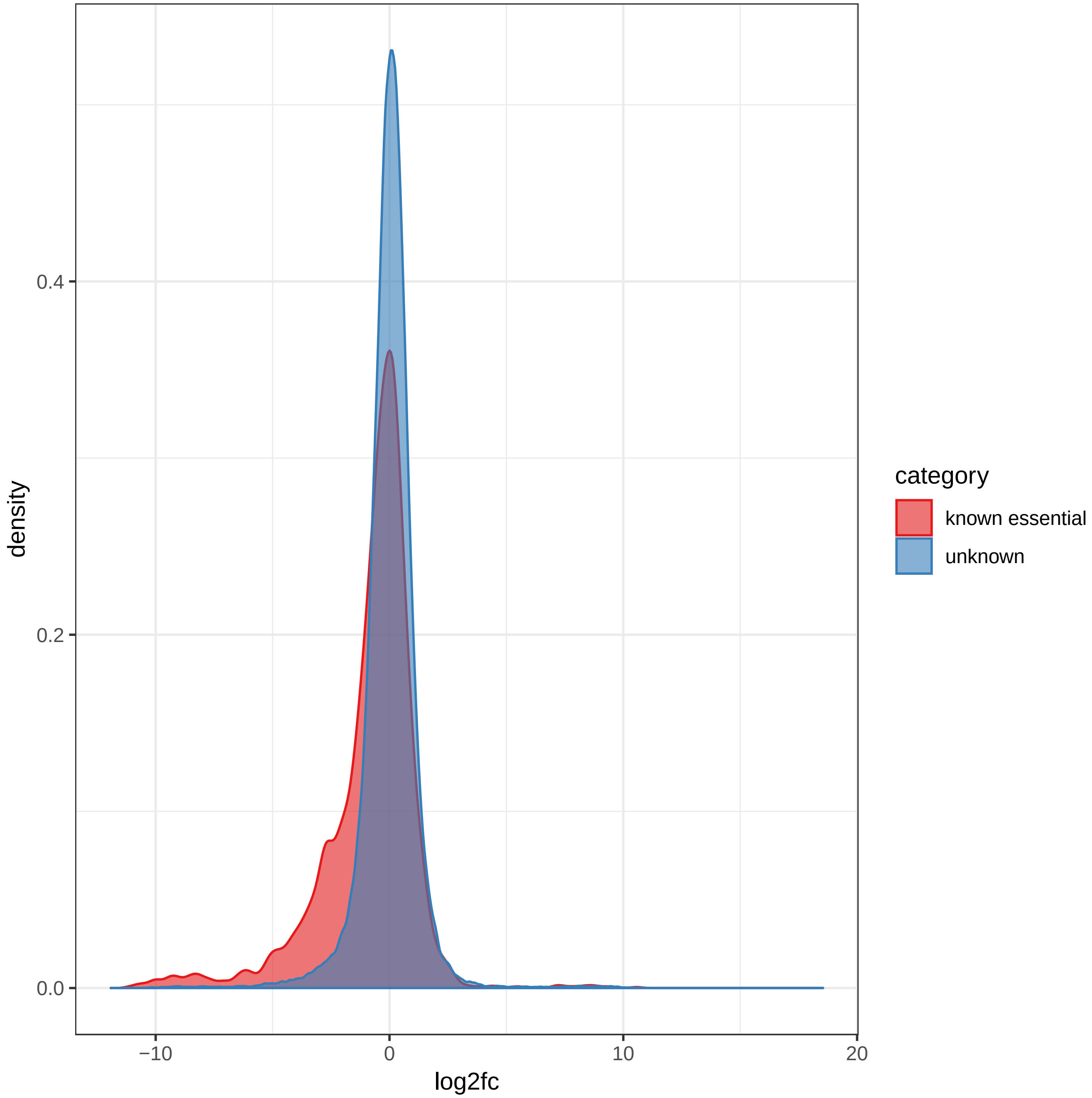

Fig S4: Distribution plots of guide-level $\log 2$ fold change for guides targeting known essential genes (red) and all other guides in the HCR116 cell line (replicate 2) from Hart et al (2015).

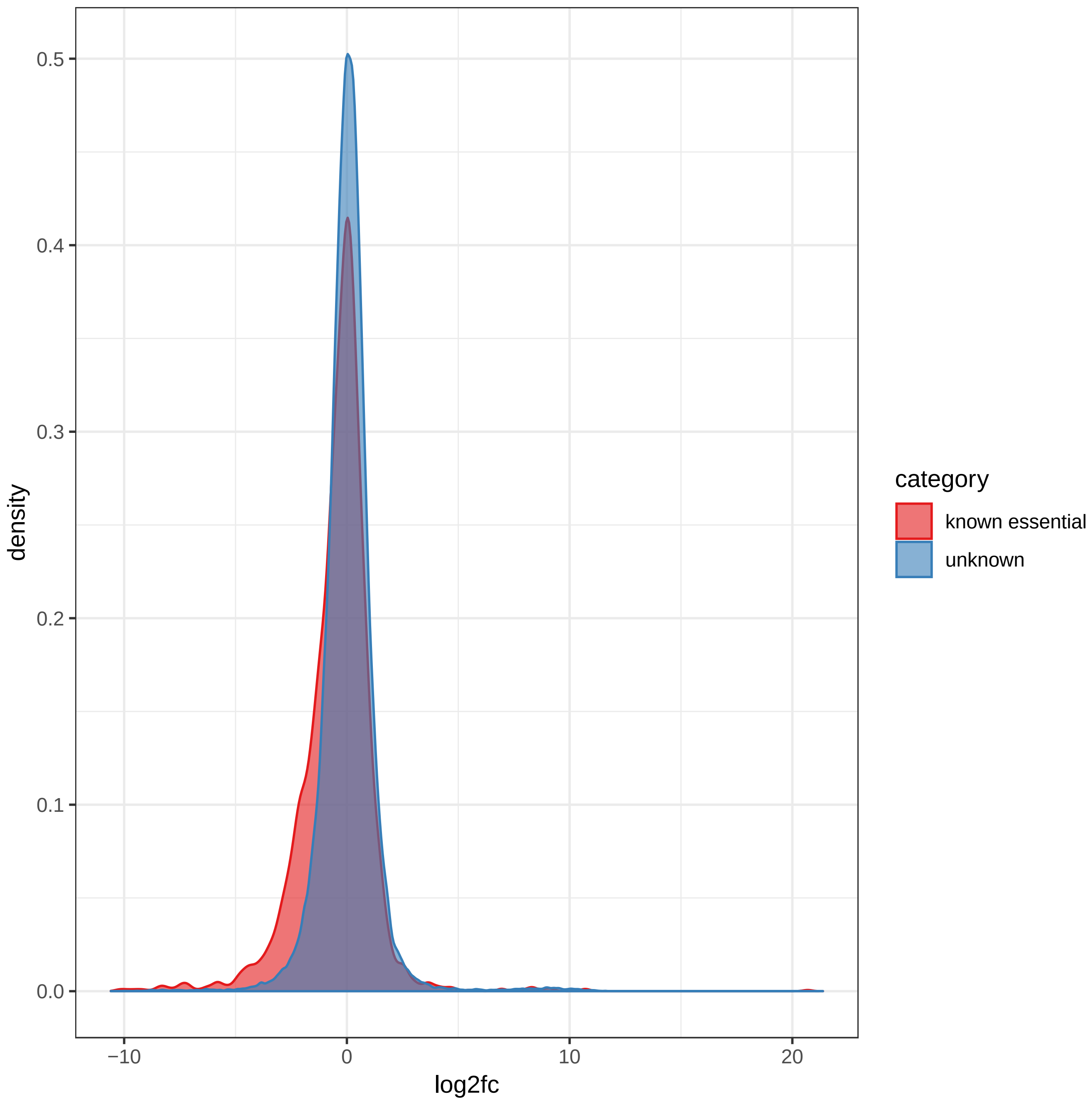

Fig S5: Distribution plots of guide-level $\log 2$ fold change for guides targeting known essential genes (red) and all other guides in the HeLa cell line from Hart et al (2015).

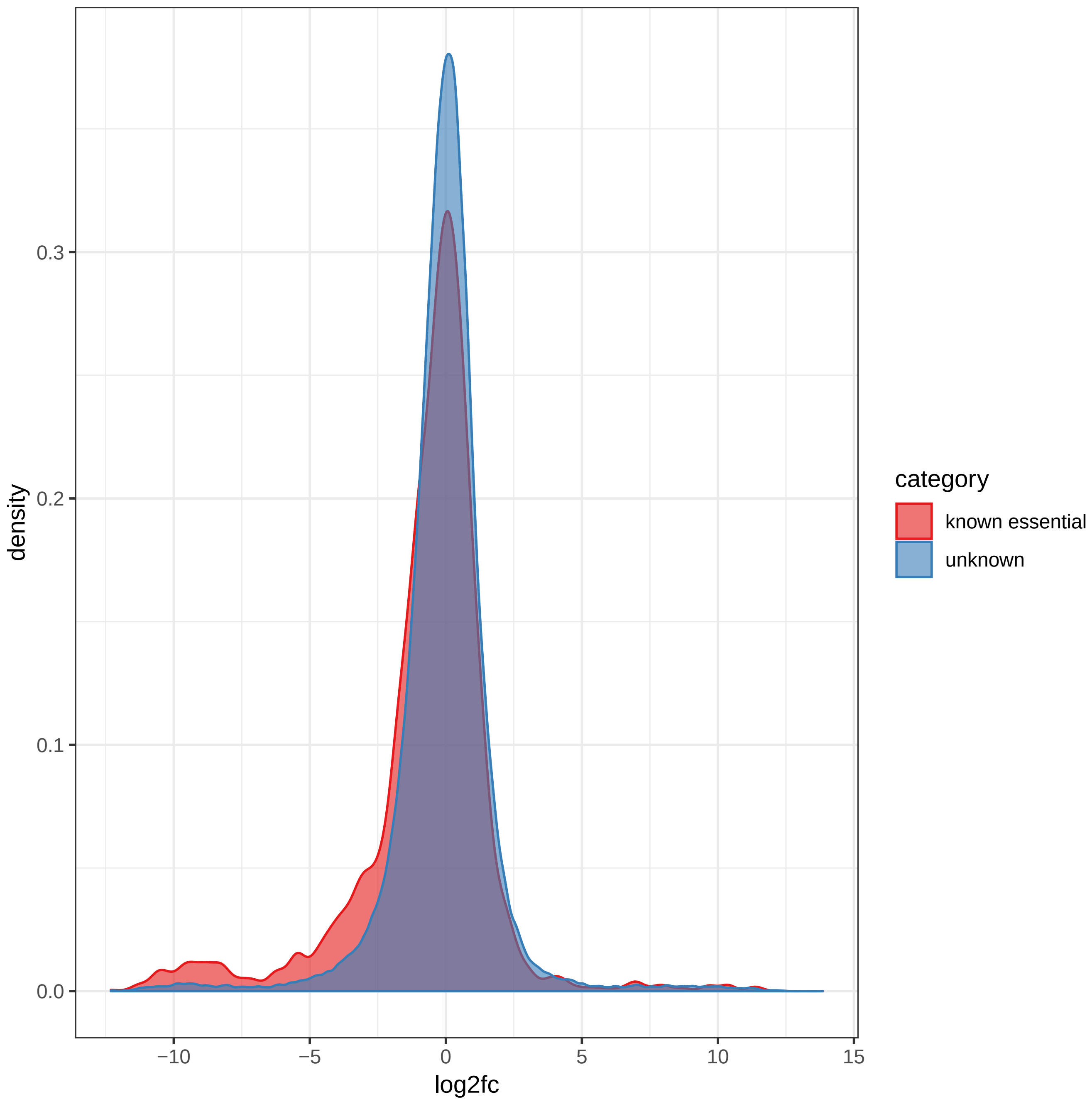

Fig S6: Distribution plots of guide-level $\log 2$ fold change for guides targeting known essential genes (red) and all other guides in the RPE1 cell line from Hart et al (2015).

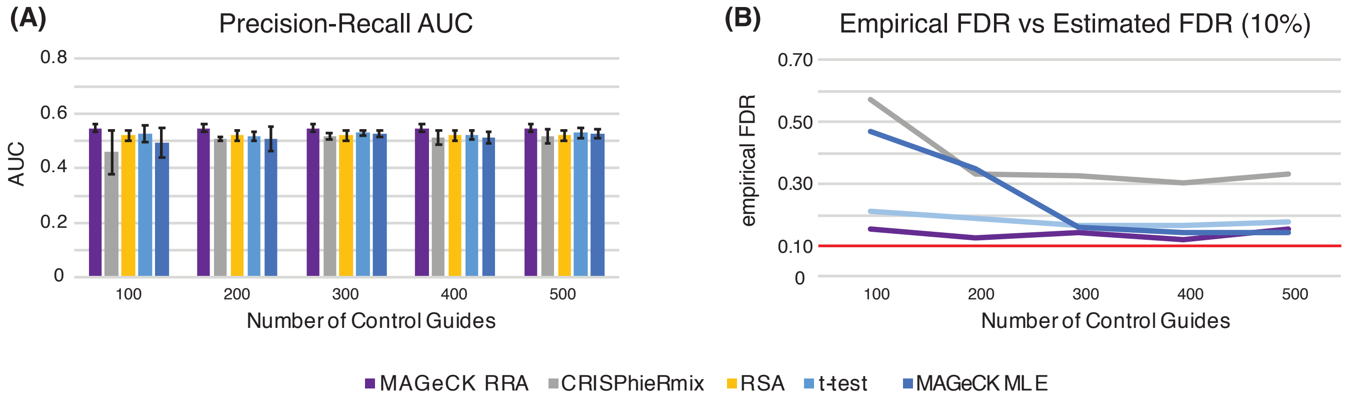

Fig S7: (A) Area under the Precision-Recall curve (PR-AUC) for each algorithm as a function of the number of control guides. The bar heights and error bars are calculated, respectively, as the mean and standard error from three replicate simulations. (B) Empirical false discovery rate at an estimated false discovery rate of 0.1. Plotted values are the average over three simulations. Missing values mean that the algorithm did not identify any significant genes at an FDR of 0.1.

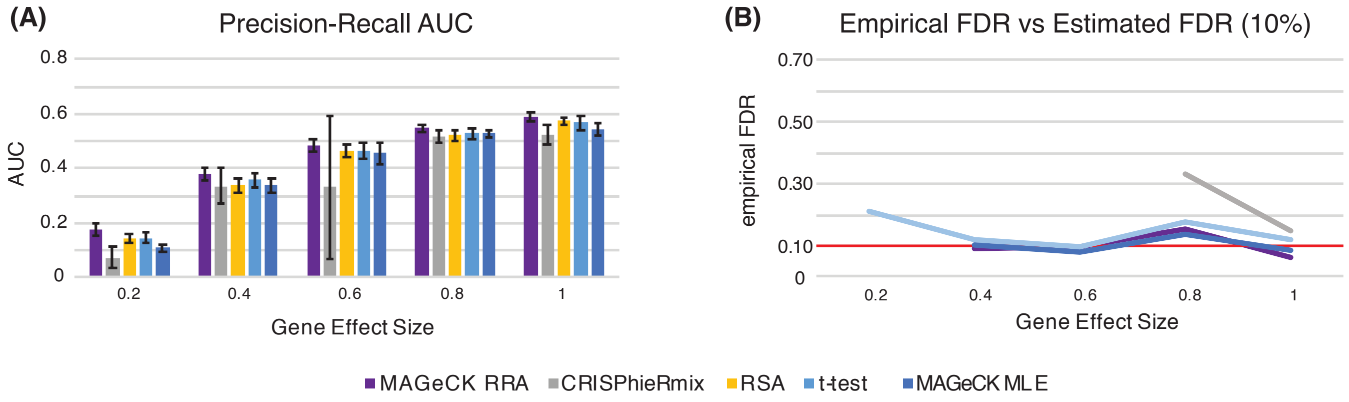

Fig S8: (A) Area under the Precision-Recall curve (PR-AUC) for each algorithm with increasing gene effect size. The gene effect size shown is the multiplier of the baseline gene effect size. The bar heights and error bars are calculated, respectively, as the mean and standard error from three replicate simulations. (B) Empirical false discovery rate at an estimated false discovery rate of 0.1. Plotted values are the average over three simulations. Missing values mean that the algorithm did not identify any significant genes at an FDR of 0.1.

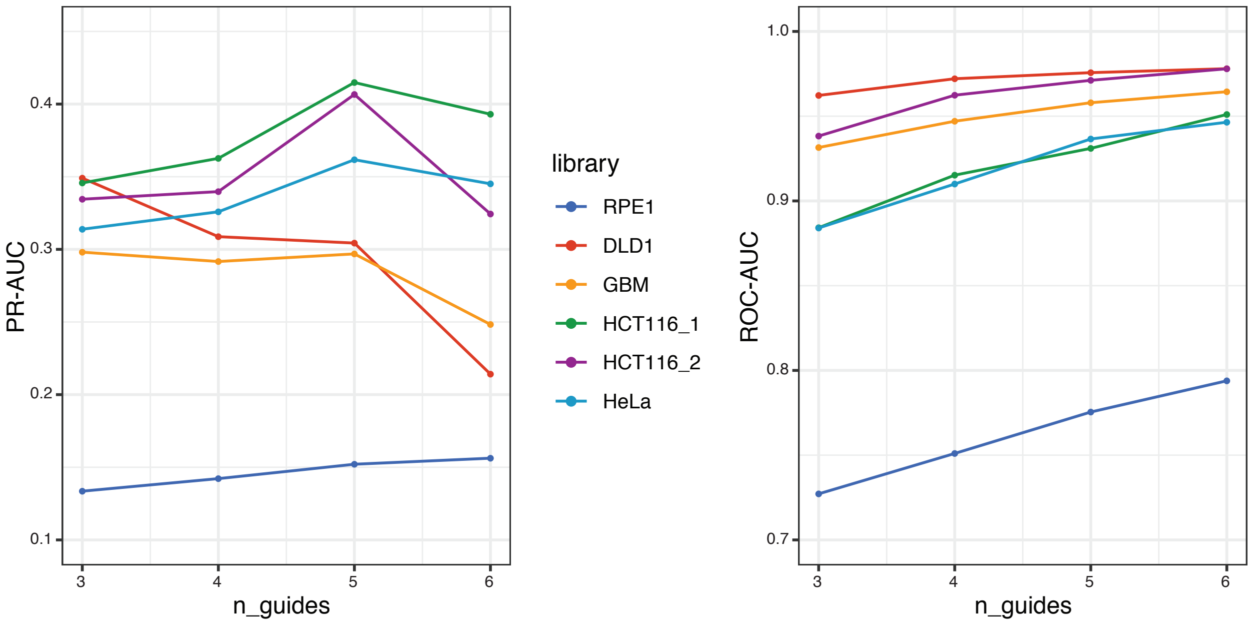

Fig S9: PR-AUC (left) and ROC-AUC (right) for TKO libraries when increasing the number of guides per gene while keeping the total sequencing depth constant.
