# Supplementary material for: A benchmark of algorithms for the analysis of pooled CRISPR screens
Source: Genome Biol. 2020 Mar 9;21:62. doi: 10.1186/s13059-020-01972-x (PMC7063732; doi:10.1186/s13059-020-01972-x)
Supplement: Supplementary file 2 — Additional file 2 Review history. [file 13059_2020_1972_MOESM2_ESM.docx]

Review History

**First round of review**

**Reviewer 1**

**Is the topic of the article timely and of interest to a wide range of readers?**

Yes

**Is the article well written and presented in a logical way?**

No: The use of English could be improved.

**Do the authors cover the relevant literature in an accurate and balanced way?**

Yes

**Do the authors provide a useful synthesis of the topic?**

No: This is a simple and straightforward "bakeoff" (i.e., benchmarking of programs). The authors do list some observations regarding the effect of numbers of guides and sequencing depth on predictive accuracy, which is useful. However, this is not a review of mathematical modeling methods for CRISPR screens, so its value in charting future directions in modeling this problem is limited.

**Do the authors provide insightful discussion of the future directions for the field?**

No

**Do any tables, figures or boxes that are present enhance the manuscript?**

Yes

**Would the manuscript benefit from any additional tables, figures or boxes?**

Yes

**Comments to author:**

This manuscript is less a review than a benchmarking paper.  A very small number of programs were compared on simulated and real data, and conclusions were drawn regarding which program performed best, and which experimental situations (read depth, number of guides per gene, effect size, etc.) resulted in higher or lower predictive accuracy.  In terms of a review, I was hoping for a more in-depth exposition of the mathematical modeling framework(s) applicable to CRISPR screens, as well as future outlook and detailed suggestions for moving forward in development of new methods.  It should also be noted that CRISPR is used not only for screens, but also for targeted editing (e.g., insertion of replacement exons, deletion of poison exons, etc.), and there are computational issues involved in validating and quantifying those edits, which are not addressed in this paper.

Several of the authors of this benchmarking paper were also the authors of one of the methods being compared (as noted in the Competing interests section).  The paper does not conclude that these authors' method performs best, however, so there should be no concerns over impartiality.  Indeed, the current paper concludes that a competing method (MAGeCK RRA) should be used in most cases.

One statement that I think could be amended is: "The number of guides needed depends on the phenotype under investigation and how strong the researchers expect the effect size will be."  Another very important factor in choosing the number of guides is the efficacy of those guides.  Some guide libraries have higher guide efficiencies (e.g., Brunello vs. Gecko), and it seems clear that more guides will be necessary when those guides have lower targeting efficiency.

In the Discussion, the authors mention several interesting issues that could probably be expanded on:

First, they discovered that a simple t-test applied to the results of DESeq2/edgeR performed particularly well.  This suggests to me that the current crop of CRISPR-screen-specific methods (e.g., MAGeCK, etc.) are not utilizing the best mathematical models, and that more work needs to go into doing a better job of modeling the data in the next generation of tools for this problem.  It would be nice if the authors could provide specific suggestions on how the mathematical modeling could be improved -- i.e., what are the most promising unexplored options for producing better predictive models for this problem?  Is the problem due to overdispersion of read counts that is not being adequately captured by existing models, or asymptotic distributional assumptions that are not valid at low sequencing depths?  Etc.

Secondly, the authors mention synergistic effects and the need for being able to detect subtle gene effects -- this is a very important point, and I'm glad the authors mentioned it.  

And lastly, the authors mention that it is better to use more guides at lower sequencing depth per guide than fewer guides with higher sequencing depth.  This is a very important observation for experimentalists, and it would be worth adding a figure that shows this tradeoff directly, perhaps by plotting the number of guides on one axis and the sequencing depth on the other -- perhaps the authors already have such a figure, but I didn't see it.

**Reviewer 2**

**Is the topic of the article timely and of interest to a wide range of readers?**

Yes

**Is the article well written and presented in a logical way?**

Yes

**Do the authors cover the relevant literature in an accurate and balanced way?**

No: see my comments to authors

**Do the authors provide a useful synthesis of the topic?**

Yes

**Do the authors provide insightful discussion of the future directions for the field?**

No

**Do any tables, figures or boxes that are present enhance the manuscript?**

Yes

**Would the manuscript benefit from any additional tables, figures or boxes?**

No: see my Comments to Author

**Comments to author:**

The authors review and benchmark a set of computational tools designed for the analysis of data from CRISPR-Cas9 pooled screens. The manuscript includes a comprehensive and nicely written background section on the CRISPR-cas9 system as well as different types of CRISPR-cas9 pooled screens, arrayed versus pooled screens, and applications of CRISPR-cas9 pooled screens toward different aims.

This is followed by listing and briefly describing the benchmarked tools as well as the simulation framework adopted in the tool comparisons, which is sensitively designed, and the considered experimental settings.  Finally, results are rigorously reported highlighting interestingly, for example that sequencing depth has a lower impact on the performances of the compared tools than previously thought and that the number of guides is more important. Moreover the authors effectively provide a final set of guidelines indicating which of the compared methods is preferable depending on variations on the different considered setting.

Given the increasing number of datasets from CRISPR-Cas9 screens being released on the public domain, together with an almost equally large number of computational tools for their analysis proposed so far, analyses and results reported in this manuscript are timely and important.

The text is well organized and the benchmarking framework is robust and well-thought.

I have a number of points that, if properly addressed, might make this manuscript suitable for publication in my opinion.

First of all, what the author do here limits to comparison of methods for the analysis of CRISRP-Cas9 pooled knock-out screen where the observed phenotype is viability reduction. Considering only 'negative' phenotype, not even sgRNA 'enrichments'. This is just a very well small subset of all the possible types of screens employing CRISPR-Cas9 that are being increasingly performed. In my opinion, title and introduction are a bit misleading in that they set the scene for a much more comprehensive comparative effort. I would suggest to tune down both and also to reduce a bit the (nice) review provided in the introduction by focusing more closely on the type of screens that have been actually benchmarked.

Furthermore, even when only considering estimating fitness effects from CRISRP-Cas9 pooled knock-out viability screen (especially those performed on cancer models) in their review, the authors should adoperate a clear distinction between three different problems with corresponding classes of tools to solve them, which have been proposed so far: 1) how to account for sgRNA on-target efficiency; 2) how to correct in-silico for gene-independent responses to CRISPR-cas9 targeting; 3) how to numerically estimate effect size and significance of gene fitness effects.

The analysis presented in this manuscript is restricted only on one problem, n. 3,  This should be clearly stated.

In addition:

* There are at least other two methods that should be cited, briefly described, and included in the first panel of table 1, alongside CERES, when describing the different approaches pursued to correct for gene-independent responses to CRISPR-cas9 targeting (putatively) arising from copy number amplifications (CNAs): CRISPRcleanR (PMID: 30103702) and the method describe in PMID:30024886. In addition, the authors should mention that CERES is not only designed for the analysis of large collection of screens (performed on heterogenous in vitro models) but it also works on a supervised way, requiring the CNA profiles of the screened models in input.
Conversely, CRISPRcleanR works in an unsupervised way (not requiring CNA profiles in input) and on a single-screen basis, in addition it is compatible and can be used upstream MAGeCK or BAGEL both.

* There is at least another important method that should be cited and briefly described (JACKS, PMID:30674557) which computes gene fitness effect significance but accounting for sgRNA on target efficiency. Particularly accounting for sgRNA on target efficiency is also a feature of CERES, which should be mentioned while describing it. We understand that like CERES, JACKs might be not steadily includable in this benchmarking effort (as it jointly analyses large collection of CRISPR-cas9 ko screens) but it should at least mentioned and briefly described.

* Importantly, the authors should specify that differently from other methods, BAGEL can be used only to estimate sgRNA depletions, i.e. gene essentialities. This is specified in Table 1 but could be mentioned while describing BAGEL.

* 'Gene effect size' should be defined at its first occurrence, i.e. while describing the simulation framework. In addition, the statistical parameter choices while simulating the baseline essential gene effects look quite arbitrary and should be justified;

* While reviewing approaches employing CRISPR-Cas9 screens to identify and prioritize new anti-cancer therapeutic targets the following works might be cited and briefly described: PMID: 30971826; PMID: 2776032;

* While mentioning cell death from excessive Cas9 induced double strand breaks in highly copy number amplified regions: it has been shown that this bias is specific to tandem duplications. This can be mentioned and the relative publication cited (PMID: 30722791)

**Authors Response**

**Point-by-point responses to the reviewers’ comments:**

*We are grateful the reviewers appreciate the need for this paper. We thank them for their helpful and insightful comments. We believe that they have helped to improve the paper tremendously. We address their comments individually below.*

Reviewer 1

This manuscript is less a review than a benchmarking paper. A very small number of programs were compared on simulated and real data, and conclusions were drawn regarding which program performed best, and which experimental situations (read depth, number of guides per gene, effect size, etc.) resulted in higher or lower predictive accuracy.

*We agree with the reviewer. We wrote it both as a review and as a benchmark. The newness of the problem of analysing CRISPR screening data means that there aren’t that many methods to compare. Our hope is that this manuscript will inspire future investigators to develop better methods. We have modified the manuscript and title to more focus on the benchmarking aspects.*

In terms of a review, I was hoping for a more in-depth exposition of the mathematical modeling framework(s) applicable to CRISPR screens, as well as future outlook and detailed suggestions for moving forward in development of new methods.

*We agree with the reviewer and have expanded the details of mathematical modeling of pooled CRISPR screening, as well as the descriptions of the algorithms for greater mathematical detail, and we have added suggestions in the discussion section on new research directions.*

It should also be noted that CRISPR is used not only for screens, but also for targeted editing (e.g., insertion of replacement exons, deletion of poison exons, etc.), and there are computational issues involved in validating and quantifying those edits, which are not addressed in this paper.

*We agree with the reviewer, and we avoided discussing this issue because we aren’t sure if the models and methods generalize. Upon the reviewers suggestion we have added a small discussion of this type of data.*

Several of the authors of this benchmarking paper were also the authors of one of the methods being compared (as noted in the Competing interests section). The paper does not conclude that these authors' method performs best, however, so there should be no concerns over impartiality. Indeed, the current paper concludes that a competing method (MAGeCK RRA) should be used in most cases.

*We thank the reviewer for pointing this out. We hope that this manuscript is impartial in its evaluation.*

One statement that I think could be amended is: "The number of guides needed depends on the phenotype under investigation and how strong the researchers expect the effect size will be." Another very important factor in choosing the number of guides is the efficacy of those guides. Some guide libraries have higher guide efficiencies (e.g., Brunello vs. Gecko), and it seems clear that more guides will be necessary when those guides have lower targeting efficiency.

*We agree with the reviewer that this is an important point and we apologize for not including it in the first version. We have added a discussion of this point in the manuscript.*

In the Discussion, the authors mention several interesting issues that could probably be expanded on:

First, they discovered that a simple t-test applied to the results of DESeq2/edgeR performed particularly well. This suggests to me that the current crop of CRISPR-screen-specific methods (e.g., MAGeCK, etc.) are not utilizing the best mathematical models, and that more work needs to go into doing a better job of modeling the data in the next generation of tools for this problem. It would be nice if the authors could provide specific suggestions on how the mathematical modeling could be improved -- i.e., what are the most promising unexplored options for producing better predictive models for this problem? Is the problem due to overdispersion of read counts that is not being adequately captured by existing models, or asymptotic distributional assumptions that are not valid at low sequencing depths? Etc.

*We thank the reviewer for their point and suggestions. We believe that the reasonable performance by the t-test is that the count modeling to account for count-specific issues (e.g. overdispersion) are modeled pretty well by existing software such as DESeq2 or edgeR. We believe that the most promising directions to investigate are specific to the experimental design of pooled CRISPR screens. For example, Imkeller et al (https://www.biorxiv.org/content/biorxiv/early/2019/08/18/699348.full.pdf) discuss the effect of the bottleneck introduced by selection and what effect this has on the distribution of log fold changes. We have included a discussion of such issues and promising directions.*

Secondly, the authors mention synergistic effects and the need for being able to detect subtle gene effects -- this is a very important point, and I'm glad the authors mentioned it.

*Thank you. We agree with the reviewer and have expanded the discussion of such issues.*

And lastly, the authors mention that it is better to use more guides at lower sequencing depth per guide than fewer guides with higher sequencing depth. This is a very important observation for experimentalists, and it would be worth adding a figure that shows this tradeoff directly, perhaps by plotting the number of guides on one axis and the sequencing depth on the other -- perhaps the authors already have such a figure, but I didn't see it.

*We agree with the reviewer that this is probably the most significant finding of our benchmarking. We have included extra analysis using the TKO data and an extra figure to help illustrate this point better.*

Reviewer 2

The authors review and benchmark a set of computational tools designed for the analysis of data from CRISPR-Cas9 pooled screens. The manuscript includes a comprehensive and nicely written background section on the CRISPR-cas9 system as well as different types of CRISPR-cas9 pooled screens, arrayed versus pooled screens, and applications of CRISPR-cas9 pooled screens toward different aims.

This is followed by listing and briefly describing the benchmarked tools as well as the simulation framework adopted in the tool comparisons, which is sensitively designed, and the considered experimental settings. Finally, results are rigorously reported highlighting interestingly, for example that sequencing depth has a lower impact on the performances of the compared tools than previously thought and that the number of guides is more important. Moreover the authors effectively provide a final set of guidelines indicating which of the compared methods is preferable depending on variations on the different considered setting.

Given the increasing number of datasets from CRISPR-Cas9 screens being released on the public domain, together with an almost equally large number of computational tools for their analysis proposed so far, analyses and results reported in this manuscript are timely and important.

The text is well organized and the benchmarking framework is robust and well-thought.

*We thank the reviewer for their kind comments.*

First of all, what the author do here limits to comparison of methods for the analysis of CRISRP-Cas9 pooled knock-out screen where the observed phenotype is viability reduction. Considering only 'negative' phenotype, not even sgRNA 'enrichments'. This is just a very well small subset of all the possible types of screens employing CRISPR-Cas9 that are being increasingly performed. In my opinion, title and introduction are a bit misleading in that they set the scene for a much more comprehensive comparative effort. I would suggest to tune down both and also to reduce a bit the (nice) review provided in the introduction by focusing more closely on the type of screens that have been actually benchmarked.

*We agree and have toned down the language and explained a bit more why we focus of negative phenotype screens. Though negative phenotype screens are a subset of possible screens, they are most prevalent and we believe that the insights gained from studying such these screens in depth are broadly applicable. Indeed, the majority of our experience with CRISPR screens come from gain of function screens, which are positive selection, and drug-based screens, where the phenotype can be both positive (drug resistance) and negative (drug susceptibility). On the other hand, the analysis of perturb-seq type screens is very different, and we expand on this point in the text.*

Furthermore, even when only considering estimating fitness effects from CRISRP-Cas9 pooled knock-out viability screen (especially those performed on cancer models) in their review, the authors should adopt a clear distinction between three different problems with corresponding classes of tools to solve them, which have been proposed so far: 1) how to account for sgRNA on-target efficiency; 2) how to correct in-silico for gene-independent responses to CRISPR-cas9 targeting; 3) how to numerically estimate effect size and significance of gene fitness effects. The analysis presented in this manuscript is restricted only on one problem, n. 3, This should be clearly stated.

*We apologize for not being clear. We hope that the text is much clearer now.

In addition, we thank the reviewer for the suggestion of papers to include in our discussion. We have included these papers and we hope that this makes the manuscript much more thorough.*

**Second round of review**

**Reviewer 2**

The authors have applied a number of edits to their main text to address the point I have raised in my previous review and clarified several points.

I have only one further minor point: the authors state that they couldn’t include JACKS in their benchmark analyses as this method was not public available when they originally performed their analysis. I think that this is borderline acceptable as JACKS was published and corresponding code public available when the authors received my comments. Nevertheless, what fairly motivates the exclusion of this method is that (like CERES) it is primarily designed to analyse multiple screens in a joint manner. The authors should amend the main text to reflect this.

**Authors Response**

**Point-by-point responses to the reviewers’ comments:**

Reviewer #2: The authors have applied a number of edits to their main text to address the point I have raised in my previous review and clarified several points.

I have only one further minor point: the authors state that they couldn’t include JACKS in their benchmark analyses as this method was not public available when they originally performed their analysis. I think that this is borderline acceptable as JACKS was published and corresponding code public available when the authors received my comments. Nevertheless, what fairly motivates the exclusion of this method is that (like CERES) it is primarily designed to analyse multiple screens in a joint manner. The authors should amend the main text to reflect this.

*Thanks for the suggestion. We have made changes following the suggestion and now the text is:*

*"Though JACKS is applicable to single screen experiments, it is primarily designed for multiple experiments where statistical pooling can help inference across all screens and therefore was not included in our single screen benchmarking."*
